# Supplementary material for: Altered microRNA Transcriptome in Cultured Human Liver Cells upon Infection with Ebola Virus
Source: Int J Mol Sci. 2021 Apr 6;22(7):3792. doi: 10.3390/ijms22073792 (PMC8038836; doi:10.3390/ijms22073792)
Supplement: Supplementary file 1 [file ijms-22-03792-s001.zip › Supplementary_File/D_ KEGG_Analysis_Results/16-30nt_pathway_Makona-24h-Huh7_vs_Control-24h-Huh7_up.mature_mirna_targets/hsa_pathwayResult.html]

| PathwayID | Definition | OriginalWebSite | Fisher-Pvalue | SelectionCounts | SelectionSize | Count | Size | FDR | Enrichment\_Score | GeneRatio | Genes |
| --- | --- | --- | --- | --- | --- | --- | --- | --- | --- | --- | --- |
| hsa05211 | Renal cell carcinoma - Homo sapiens (human) | http://www.genome.jp/kegg-bin/show\_pathway?hsa05211+1399+54583+112399+2033+2113+3725+5594+4233+56924+5290+5291+5894+5906+5908+6655+7030 | 2.532827e-07 | 16 | 404 | 69 | 7906 | 8.459641e-05 | 6.596395 | 0.039604 | CRKL//EGLN1//EGLN3//EP300//ETS1//JUN//MAPK1//MET//PAK6//PIK3CA//PIK3CB//RAF1//RAP1A//RAP1B//SOS2//TFE3 |
| hsa04350 | TGF-beta signaling pathway - Homo sapiens (human) | http://www.genome.jp/kegg-bin/show\_pathway?hsa04350+90+25805+659+1875+2033+8200+3398+3458+3625+5594+9241+5515+5516+6500+57154+7048+7050+7124 | 9.616020e-07 | 18 | 404 | 94 | 7906 | 1.605875e-04 | 6.017005 | 0.044554 | ACVR1//BAMBI//BMPR2//E2F5//EP300//GDF5//ID2//IFNG//INHBB//MAPK1//NOG//PPP2CA//PPP2CB//SKP1//SMURF1//TGFBR2//TGIF1//TNF |
| hsa04068 | FoxO signaling pathway - Homo sapiens (human) | http://www.genome.jp/kegg-bin/show\_pathway?hsa04068+100533105+595+894+2033+23710+1647+9456+3479+5594+5290+5291+5562+5728+5894+1901+6446+10110+23678+23411+6655+7048 | 2.566090e-06 | 21 | 404 | 131 | 7906 | 2.856914e-04 | 5.590728 | 0.051980 | C8ORF44-SGK3//CCND1//CCND2//EP300//GABARAPL1//GADD45A//HOMER1//IGF1//MAPK1//PIK3CA//PIK3CB//PRKAA1//PTEN//RAF1//S1PR1//SGK1//SGK2//SGK3//SIRT1//SOS2//TGFBR2 |
| hsa05225 | Hepatocellular carcinoma - Homo sapiens (human) | http://www.genome.jp/kegg-bin/show\_pathway?hsa05225+196528+595+7976+8323+1647+2939+2947+4040+5594+4233+4257+5290+5291+5728+5894+5925+6595+6603+6655+7048+80326+7473+7476 | 1.331982e-05 | 23 | 404 | 168 | 7906 | 9.075104e-04 | 4.875502 | 0.056931 | ARID2//CCND1//FZD3//FZD6//GADD45A//GSTA2//GSTM3//LRP6//MAPK1//MET//MGST1//PIK3CA//PIK3CB//PTEN//RAF1//RB1//SMARCA2//SMARCD2//SOS2//TGFBR2//WNT10A//WNT3//WNT7A |
| hsa05200 | Pathways in cancer - Homo sapiens (human) | http://www.genome.jp/kegg-bin/show\_pathway?hsa05200+109+26060+367+805+8030+595+894+1399+28514+1906+1910+54583+112399+2033+2099+2113+7976+8323+1647+2737+2776+2939+2947+3439+3458+3479+3558+3725+1902+4040+5594+4233+4257+5290+5291+5366+5468+5567+5728+5894+10928+5925+9252+6500+6655+7048+7423+80326+7473+7476 | 1.358548e-05 | 50 | 404 | 529 | 7906 | 9.075104e-04 | 4.866925 | 0.123762 | ADCY3//APPL1//AR//CALM2//CCDC6//CCND1//CCND2//CRKL//DLL1//EDN1//EDNRB//EGLN1//EGLN3//EP300//ESR1//ETS1//FZD3//FZD6//GADD45A//GLI3//GNAQ//GSTA2//GSTM3//IFNA1//IFNG//IGF1//IL2//JUN//LPAR1//LRP6//MAPK1//MET//MGST1//PIK3CA//PIK3CB//PMAIP1//PPARG//PRKACB//PTEN//RAF1//RALBP1//RB1//RPS6KA5//SKP1//SOS2//TGFBR2//VEGFB//WNT10A//WNT3//WNT7A |
| hsa04150 | mTOR signaling pathway - Homo sapiens (human) | http://www.genome.jp/kegg-bin/show\_pathway?hsa04150+6249+7976+8323+3479+4040+5594+5290+5291+5562+5728+5894+253260+58528+6446+6655+7124+7248+9706+80326+7473+7476 | 3.042493e-05 | 21 | 404 | 153 | 7906 | 1.693654e-03 | 4.516770 | 0.051980 | CLIP1//FZD3//FZD6//IGF1//LRP6//MAPK1//PIK3CA//PIK3CB//PRKAA1//PTEN//RAF1//RICTOR//RRAGD//SGK1//SOS2//TNF//TSC1//ULK2//WNT10A//WNT3//WNT7A |
| hsa04360 | Axon guidance - Homo sapiens (human) | http://www.genome.jp/kegg-bin/show\_pathway?hsa04360+659+1073+1808+1942+1948+55740+7976+5594+4233+4690+56924+5290+5291+91584+5534+5535+5894+5921+6092+54910+9353+7222+219699 | 4.493441e-05 | 23 | 404 | 181 | 7906 | 2.144013e-03 | 4.347421 | 0.056931 | BMPR2//CFL2//DPYSL2//EFNA1//EFNB2//ENAH//FZD3//MAPK1//MET//NCK1//PAK6//PIK3CA//PIK3CB//PLXNA4//PPP3R1//PPP3R2//RAF1//RASA1//ROBO2//SEMA4C//SLIT2//TRPC3//UNC5B |
| hsa05224 | Breast cancer - Homo sapiens (human) | http://www.genome.jp/kegg-bin/show\_pathway?hsa05224+595+28514+2099+7976+8323+1647+3479+3725+4040+5594+5290+5291+5728+5894+5925+6655+80326+7473+7476 | 1.639036e-04 | 19 | 404 | 147 | 7906 | 5.092957e-03 | 3.785411 | 0.047030 | CCND1//DLL1//ESR1//FZD3//FZD6//GADD45A//IGF1//JUN//LRP6//MAPK1//PIK3CA//PIK3CB//PTEN//RAF1//RB1//SOS2//WNT10A//WNT3//WNT7A |
| hsa04218 | Cellular senescence - Homo sapiens (human) | http://www.genome.jp/kegg-bin/show\_pathway?hsa04218+805+595+894+1875+2113+1647+10114+3486+5594+5290+5291+5534+5535+5728+5894+5925+23411+293+7048+7248 | 1.762652e-04 | 20 | 404 | 160 | 7906 | 5.092957e-03 | 3.753833 | 0.049505 | CALM2//CCND1//CCND2//E2F5//ETS1//GADD45A//HIPK3//IGFBP3//MAPK1//PIK3CA//PIK3CB//PPP3R1//PPP3R2//PTEN//RAF1//RB1//SIRT1//SLC25A6//TGFBR2//TSC1 |
| hsa04310 | Wnt signaling pathway - Homo sapiens (human) | http://www.genome.jp/kegg-bin/show\_pathway?hsa04310+25805+595+894+80319+2033+8061+7976+8323+3725+4040+5534+5535+166336+5567+4919+6500+79718+80326+7473+7476 | 1.762652e-04 | 20 | 404 | 160 | 7906 | 5.092957e-03 | 3.753833 | 0.049505 | BAMBI//CCND1//CCND2//CXXC4//EP300//FOSL1//FZD3//FZD6//JUN//LRP6//PPP3R1//PPP3R2//PRICKLE2//PRKACB//ROR1//SKP1//TBL1XR1//WNT10A//WNT3//WNT7A |
| hsa05202 | Transcriptional misregulation in cancer - Homo sapiens (human) | http://www.genome.jp/kegg-bin/show\_pathway?hsa05202+4299+597+894+905+1051+2119+2313+1647+3398+3479+3486+51274+4094+4233+4613+5079+5468+6760+7030+7048+7490+6935 | 1.912492e-04 | 22 | 404 | 186 | 7906 | 5.092957e-03 | 3.718400 | 0.054455 | AFF1//BCL2A1//CCND2//CCNT2//CEBPB//ETV5//FLI1//GADD45A//ID2//IGF1//IGFBP3//KLF3//MAF//MET//MYCN//PAX5//PPARG//SS18//TFE3//TGFBR2//WT1//ZEB1 |
| hsa04917 | Prolactin signaling pathway - Homo sapiens (human) | http://www.genome.jp/kegg-bin/show\_pathway?hsa04917+595+894+2099+3659+5594+5290+5291+5894+8651+9021+9655+6655 | 1.928447e-04 | 12 | 404 | 70 | 7906 | 5.092957e-03 | 3.714792 | 0.029703 | CCND1//CCND2//ESR1//IRF1//MAPK1//PIK3CA//PIK3CB//RAF1//SOCS1//SOCS3//SOCS5//SOS2 |
| hsa04660 | T cell receptor signaling pathway - Homo sapiens (human) | http://www.genome.jp/kegg-bin/show\_pathway?hsa04660+916+925+3458+3558+3725+5594+4690+56924+5290+5291+5534+5535+5894+6655+7124 | 2.133321e-04 | 15 | 404 | 103 | 7906 | 5.092957e-03 | 3.670944 | 0.037129 | CD3E//CD8A//IFNG//IL2//JUN//MAPK1//NCK1//PAK6//PIK3CA//PIK3CB//PPP3R1//PPP3R2//RAF1//SOS2//TNF |
| hsa05418 | Fluid shear stress and atherosclerosis - Homo sapiens (human) | http://www.genome.jp/kegg-bin/show\_pathway?hsa05418+90+659+805+6347+1843+1906+2939+2947+3458+3725+3791+4205+4257+5290+5291+5562+6382+7124 | 2.367818e-04 | 18 | 404 | 139 | 7906 | 5.092957e-03 | 3.625652 | 0.044554 | ACVR1//BMPR2//CALM2//CCL2//DUSP1//EDN1//GSTA2//GSTM3//IFNG//JUN//KDR//MEF2A//MGST1//PIK3CA//PIK3CB//PRKAA1//SDC1//TNF |
| hsa04115 | p53 signaling pathway - Homo sapiens (human) | http://www.genome.jp/kegg-bin/show\_pathway?hsa04115+595+894+9538+1647+3479+3486+4194+5366+5728+27244+10572+64393 | 2.538643e-04 | 12 | 404 | 72 | 7906 | 5.092957e-03 | 3.595398 | 0.029703 | CCND1//CCND2//EI24//GADD45A//IGF1//IGFBP3//MDM4//PMAIP1//PTEN//SESN1//SIVA1//ZMAT3 |
| hsa04114 | Oocyte meiosis - Homo sapiens (human) | http://www.genome.jp/kegg-bin/show\_pathway?hsa04114+109+367+805+64506+132864+22849+3479+5594+5515+5516+5529+5534+5535+5567+6500+9748+7532 | 2.586852e-04 | 17 | 404 | 128 | 7906 | 5.092957e-03 | 3.587228 | 0.042079 | ADCY3//AR//CALM2//CPEB1//CPEB2//CPEB3//IGF1//MAPK1//PPP2CA//PPP2CB//PPP2R5E//PPP3R1//PPP3R2//PRKACB//SKP1//SLK//YWHAG |
| hsa04550 | Signaling pathways regulating pluripotency of stem cells - Homo sapiens (human) | http://www.genome.jp/kegg-bin/show\_pathway?hsa04550+90+659+7976+8323+3398+3479+3625+3670+3720+5594+4762+84333+5290+5291+5894+80326+7473+7476 | 2.592223e-04 | 18 | 404 | 140 | 7906 | 5.092957e-03 | 3.586328 | 0.044554 | ACVR1//BMPR2//FZD3//FZD6//ID2//IGF1//INHBB//ISL1//JARID2//MAPK1//NEUROG1//PCGF5//PIK3CA//PIK3CB//RAF1//WNT10A//WNT3//WNT7A |
| hsa04916 | Melanogenesis - Homo sapiens (human) | http://www.genome.jp/kegg-bin/show\_pathway?hsa04916+109+805+1906+1910+2033+7976+8323+2776+5594+5567+5894+80326+7473+7476 | 5.766433e-04 | 14 | 404 | 101 | 7906 | 1.069994e-02 | 3.239093 | 0.034653 | ADCY3//CALM2//EDN1//EDNRB//EP300//FZD3//FZD6//GNAQ//MAPK1//PRKACB//RAF1//WNT10A//WNT3//WNT7A |
| hsa05142 | Chagas disease (American trypanosomiasis) - Homo sapiens (human) | http://www.genome.jp/kegg-bin/show\_pathway?hsa05142+6347+916+2776+3458+3558+51135+3725+5594+5290+5291+5515+5516+7048+7124 | 6.379967e-04 | 14 | 404 | 102 | 7906 | 1.121531e-02 | 3.195182 | 0.034653 | CCL2//CD3E//GNAQ//IFNG//IL2//IRAK4//JUN//MAPK1//PIK3CA//PIK3CB//PPP2CA//PPP2CB//TGFBR2//TNF |
| hsa04710 | Circadian rhythm - Homo sapiens (human) | http://www.genome.jp/kegg-bin/show\_pathway?hsa04710+8553+79365+9575+4862+5562+6095+6500 | 7.802292e-04 | 7 | 404 | 31 | 7906 | 1.302983e-02 | 3.107778 | 0.017327 | BHLHE40//BHLHE41//CLOCK//NPAS2//PRKAA1//RORA//SKP1 |
| hsa04934 | Cushing syndrome - Homo sapiens (human) | http://www.genome.jp/kegg-bin/show\_pathway?hsa04934+109+595+1394+7976+8323+2776+3739+3776+3949+5594+5567+5906+5908+51655+5925+80326+7473+7476 | 8.973753e-04 | 18 | 404 | 155 | 7906 | 1.427254e-02 | 3.047026 | 0.044554 | ADCY3//CCND1//CRHR1//FZD3//FZD6//GNAQ//KCNA4//KCNK2//LDLR//MAPK1//PRKACB//RAP1A//RAP1B//RASD1//RB1//WNT10A//WNT3//WNT7A |
| hsa04510 | Focal adhesion - Homo sapiens (human) | http://www.genome.jp/kegg-bin/show\_pathway?hsa04510+595+894+1399+2316+3479+3696+3725+3791+5594+4233+56924+5290+5291+4659+5728+5894+5906+5908+6655+7423+7791 | 1.218884e-03 | 21 | 404 | 199 | 7906 | 1.770411e-02 | 2.914037 | 0.051980 | CCND1//CCND2//CRKL//FLNA//IGF1//ITGB8//JUN//KDR//MAPK1//MET//PAK6//PIK3CA//PIK3CB//PPP1R12A//PTEN//RAF1//RAP1A//RAP1B//SOS2//VEGFB//ZYX |
| hsa05215 | Prostate cancer - Homo sapiens (human) | http://www.genome.jp/kegg-bin/show\_pathway?hsa05215+367+595+2033+2119+3479+5594+5290+5291+5728+5894+5925+6655+6935 | 1.237779e-03 | 13 | 404 | 97 | 7906 | 1.770411e-02 | 2.907357 | 0.032178 | AR//CCND1//EP300//ETV5//IGF1//MAPK1//PIK3CA//PIK3CB//PTEN//RAF1//RB1//SOS2//ZEB1 |
| hsa05210 | Colorectal cancer - Homo sapiens (human) | http://www.genome.jp/kegg-bin/show\_pathway?hsa05210+26060+595+2069+1647+3725+5594+5290+5291+5366+5894+6655+7048 | 1.324091e-03 | 12 | 404 | 86 | 7906 | 1.770411e-02 | 2.878082 | 0.029703 | APPL1//CCND1//EREG//GADD45A//JUN//MAPK1//PIK3CA//PIK3CB//PMAIP1//RAF1//SOS2//TGFBR2 |
| hsa01522 | Endocrine resistance - Homo sapiens (human) | http://www.genome.jp/kegg-bin/show\_pathway?hsa01522+109+595+28514+2099+3479+3725+5594+5290+5291+5567+5894+5925+6655 | 1.362586e-03 | 13 | 404 | 98 | 7906 | 1.770411e-02 | 2.865636 | 0.032178 | ADCY3//CCND1//DLL1//ESR1//IGF1//JUN//MAPK1//PIK3CA//PIK3CB//PRKACB//RAF1//RB1//SOS2 |
| hsa05214 | Glioma - Homo sapiens (human) | http://www.genome.jp/kegg-bin/show\_pathway?hsa05214+805+595+1647+3479+5594+5290+5291+5728+5894+5925+6655 | 1.378165e-03 | 11 | 404 | 75 | 7906 | 1.770411e-02 | 2.860699 | 0.027228 | CALM2//CCND1//GADD45A//IGF1//MAPK1//PIK3CA//PIK3CB//PTEN//RAF1//RB1//SOS2 |
| hsa04140 | Autophagy - animal - Homo sapiens (human) | http://www.genome.jp/kegg-bin/show\_pathway?hsa04140+55054+23710+5594+29982+5290+5291+5515+5516+5562+5567+5728+5894+58528+29110+7248+9706 | 1.606995e-03 | 16 | 404 | 137 | 7906 | 1.987912e-02 | 2.793985 | 0.039604 | ATG16L1//GABARAPL1//MAPK1//NRBF2//PIK3CA//PIK3CB//PPP2CA//PPP2CB//PRKAA1//PRKACB//PTEN//RAF1//RRAGD//TBK1//TSC1//ULK2 |
| hsa05205 | Proteoglycans in cancer - Homo sapiens (human) | http://www.genome.jp/kegg-bin/show\_pathway?hsa05205+595+2099+2316+7976+8323+3479+3791+5594+4233+4478+5290+5291+4659+5567+5894+6382+6655+7124+80326+7473+7476 | 1.667097e-03 | 21 | 404 | 204 | 7906 | 1.988609e-02 | 2.778039 | 0.051980 | CCND1//ESR1//FLNA//FZD3//FZD6//IGF1//KDR//MAPK1//MET//MSN//PIK3CA//PIK3CB//PPP1R12A//PRKACB//RAF1//SDC1//SOS2//TNF//WNT10A//WNT3//WNT7A |
| hsa04211 | Longevity regulating pathway - Homo sapiens (human) | http://www.genome.jp/kegg-bin/show\_pathway?hsa04211+109+79602+26060+3479+5290+5291+5468+5562+5567+27244+23411+7248 | 1.790572e-03 | 12 | 404 | 89 | 7906 | 2.062245e-02 | 2.747008 | 0.029703 | ADCY3//ADIPOR2//APPL1//IGF1//PIK3CA//PIK3CB//PPARG//PRKAA1//PRKACB//SESN1//SIRT1//TSC1 |
| hsa04720 | Long-term potentiation - Homo sapiens (human) | http://www.genome.jp/kegg-bin/show\_pathway?hsa04720+805+2033+2776+5594+5534+5535+5567+5894+5906+5908 | 1.969454e-03 | 10 | 404 | 67 | 7906 | 2.192659e-02 | 2.705654 | 0.024752 | CALM2//EP300//GNAQ//MAPK1//PPP3R1//PPP3R2//PRKACB//RAF1//RAP1A//RAP1B |
| hsa04390 | Hippo signaling pathway - Homo sapiens (human) | http://www.genome.jp/kegg-bin/show\_pathway?hsa04390+659+595+894+122786+7976+8323+8200+3398+92597+5515+5516+8463+7048+80326+7473+7476+7532 | 2.154759e-03 | 17 | 404 | 154 | 7906 | 2.204069e-02 | 2.666601 | 0.042079 | BMPR2//CCND1//CCND2//FRMD6//FZD3//FZD6//GDF5//ID2//MOB1B//PPP2CA//PPP2CB//TEAD2//TGFBR2//WNT10A//WNT3//WNT7A//YWHAG |
| hsa04151 | PI3K-Akt signaling pathway - Homo sapiens (human) | http://www.genome.jp/kegg-bin/show\_pathway?hsa04151+100533105+595+894+1129+1942+2069+3439+3479+3558+3696+3791+1902+5594+4233+4602+4908+5290+5291+5515+5516+5529+5562+5728+5894+6446+10110+23678+6655+7248+7423+7532 | 2.207655e-03 | 31 | 404 | 354 | 7906 | 2.204069e-02 | 2.656069 | 0.076733 | C8ORF44-SGK3//CCND1//CCND2//CHRM2//EFNA1//EREG//IFNA1//IGF1//IL2//ITGB8//KDR//LPAR1//MAPK1//MET//MYB//NTF3//PIK3CA//PIK3CB//PPP2CA//PPP2CB//PPP2R5E//PRKAA1//PTEN//RAF1//SGK1//SGK2//SGK3//SOS2//TSC1//VEGFB//YWHAG |
| hsa04010 | MAPK signaling pathway - Homo sapiens (human) | http://www.genome.jp/kegg-bin/show\_pathway?hsa04010+1399+1843+1942+2069+2316+1647+3479+51135+3725+3791+5594+4233+4908+5534+5535+5567+5894+5906+5908+9693+5921+9252+6655+57551+7048+7124+7423 | 2.256363e-03 | 27 | 404 | 295 | 7906 | 2.204069e-02 | 2.646591 | 0.066832 | CRKL//DUSP1//EFNA1//EREG//FLNA//GADD45A//IGF1//IRAK4//JUN//KDR//MAPK1//MET//NTF3//PPP3R1//PPP3R2//PRKACB//RAF1//RAP1A//RAP1B//RAPGEF2//RASA1//RPS6KA5//SOS2//TAOK1//TGFBR2//TNF//VEGFB |
| hsa05206 | MicroRNAs in cancer - Homo sapiens (human) | http://www.genome.jp/kegg-bin/show\_pathway?hsa05206+659+595+894+1399+1789+1942+2033+27086+7976+5594+4082+4194+4233+5290+5291+5728+5894+8434+9252+23411+8651+6655+6659+8626+7329+7473+6935+23414 | 2.285404e-03 | 28 | 404 | 310 | 7906 | 2.204069e-02 | 2.641037 | 0.069307 | BMPR2//CCND1//CCND2//CRKL//DNMT3B//EFNA1//EP300//FOXP1//FZD3//MAPK1//MARCKS//MDM4//MET//PIK3CA//PIK3CB//PTEN//RAF1//RECK//RPS6KA5//SIRT1//SOCS1//SOS2//SOX4//TP63//UBE2I//WNT3//ZEB1//ZFPM2 |
| hsa05160 | Hepatitis C - Homo sapiens (human) | http://www.genome.jp/kegg-bin/show\_pathway?hsa05160+595+3439+3458+3949+5594+57506+5290+5291+5515+5516+5894+5925+9021+6655+29110+7124+7532 | 2.309654e-03 | 17 | 404 | 155 | 7906 | 2.204069e-02 | 2.636453 | 0.042079 | CCND1//IFNA1//IFNG//LDLR//MAPK1//MAVS//PIK3CA//PIK3CB//PPP2CA//PPP2CB//RAF1//RB1//SOCS3//SOS2//TBK1//TNF//YWHAG |
| hsa04924 | Renin secretion - Homo sapiens (human) | http://www.genome.jp/kegg-bin/show\_pathway?hsa04924+116+153+805+1906+1907+2776+3759+5534+5535+5567 | 2.465971e-03 | 10 | 404 | 69 | 7906 | 2.287873e-02 | 2.608012 | 0.024752 | ADCYAP1//ADRB1//CALM2//EDN1//EDN2//GNAQ//KCNJ2//PPP3R1//PPP3R2//PRKACB |
| hsa04024 | cAMP signaling pathway - Homo sapiens (human) | http://www.genome.jp/kegg-bin/show\_pathway?hsa04024+109+116+153+805+1129+1261+1906+1907+2033+2737+3725+5594+5290+5291+4659+84152+5567+5894+5906+5908+11069 | 3.345093e-03 | 21 | 404 | 216 | 7906 | 2.983244e-02 | 2.475592 | 0.051980 | ADCY3//ADCYAP1//ADRB1//CALM2//CHRM2//CNGA3//EDN1//EDN2//EP300//GLI3//JUN//MAPK1//PIK3CA//PIK3CB//PPP1R12A//PPP1R1B//PRKACB//RAF1//RAP1A//RAP1B//RAPGEF4 |
| hsa05218 | Melanoma - Homo sapiens (human) | http://www.genome.jp/kegg-bin/show\_pathway?hsa05218+595+1647+3479+5594+4233+5290+5291+5728+5894+5925 | 3.394110e-03 | 10 | 404 | 72 | 7906 | 2.983244e-02 | 2.469274 | 0.024752 | CCND1//GADD45A//IGF1//MAPK1//MET//PIK3CA//PIK3CB//PTEN//RAF1//RB1 |
| hsa04014 | Ras signaling pathway - Homo sapiens (human) | http://www.genome.jp/kegg-bin/show\_pathway?hsa04014+805+1942+2113+3479+3791+5594+4233+4908+56924+5290+5291+5567+5894+10928+5906+5908+5921+23179+6655+8831+29110+7423 | 3.667894e-03 | 22 | 404 | 232 | 7906 | 3.096688e-02 | 2.435583 | 0.054455 | CALM2//EFNA1//ETS1//IGF1//KDR//MAPK1//MET//NTF3//PAK6//PIK3CA//PIK3CB//PRKACB//RAF1//RALBP1//RAP1A//RAP1B//RASA1//RGL1//SOS2//SYNGAP1//TBK1//VEGFB |
| hsa05226 | Gastric cancer - Homo sapiens (human) | http://www.genome.jp/kegg-bin/show\_pathway?hsa05226+595+7976+8323+1647+4040+5594+4233+5290+5291+5894+5925+6655+7048+80326+7473+7476 | 3.798216e-03 | 16 | 404 | 149 | 7906 | 3.096688e-02 | 2.420420 | 0.039604 | CCND1//FZD3//FZD6//GADD45A//LRP6//MAPK1//MET//PIK3CA//PIK3CB//RAF1//RB1//SOS2//TGFBR2//WNT10A//WNT3//WNT7A |
| hsa04012 | ErbB signaling pathway - Homo sapiens (human) | http://www.genome.jp/kegg-bin/show\_pathway?hsa04012+1399+2069+3725+5594+4690+3084+56924+5290+5291+5894+6655 | 3.801324e-03 | 11 | 404 | 85 | 7906 | 3.096688e-02 | 2.420065 | 0.027228 | CRKL//EREG//JUN//MAPK1//NCK1//NRG1//PAK6//PIK3CA//PIK3CB//RAF1//SOS2 |
| hsa05231 | Choline metabolism in cancer - Homo sapiens (human) | http://www.genome.jp/kegg-bin/show\_pathway?hsa05231+8526+160851+56261+3725+5594+5290+5291+5894+23446+6655+7248+10810 | 4.053558e-03 | 12 | 404 | 98 | 7906 | 3.223543e-02 | 2.392164 | 0.029703 | DGKE//DGKH//GPCPD1//JUN//MAPK1//PIK3CA//PIK3CB//RAF1//SLC44A1//SOS2//TSC1//WASF3 |
| hsa05220 | Chronic myeloid leukemia - Homo sapiens (human) | http://www.genome.jp/kegg-bin/show\_pathway?hsa05220+595+1399+1647+5594+5290+5291+5894+5925+6655+7048 | 5.041414e-03 | 10 | 404 | 76 | 7906 | 3.915889e-02 | 2.297448 | 0.024752 | CCND1//CRKL//GADD45A//MAPK1//PIK3CA//PIK3CB//RAF1//RB1//SOS2//TGFBR2 |
| hsa04015 | Rap1 signaling pathway - Homo sapiens (human) | http://www.genome.jp/kegg-bin/show\_pathway?hsa04015+109+805+1399+1942+55740+2776+3479+3791+1902+5594+4233+5217+5290+5291+5894+5906+5908+9693+11069+7423 | 5.223469e-03 | 20 | 404 | 210 | 7906 | 3.965088e-02 | 2.282041 | 0.049505 | ADCY3//CALM2//CRKL//EFNA1//ENAH//GNAQ//IGF1//KDR//LPAR1//MAPK1//MET//PFN2//PIK3CA//PIK3CB//RAF1//RAP1A//RAP1B//RAPGEF2//RAPGEF4//VEGFB |
| hsa04380 | Osteoclast differentiation - Homo sapiens (human) | http://www.genome.jp/kegg-bin/show\_pathway?hsa04380+8061+3458+3725+5594+5290+5291+5468+5534+5535+140885+8651+9021+7048+7124 | 5.591757e-03 | 14 | 404 | 128 | 7906 | 4.150326e-02 | 2.252452 | 0.034653 | FOSL1//IFNG//JUN//MAPK1//PIK3CA//PIK3CB//PPARG//PPP3R1//PPP3R2//SIRPA//SOCS1//SOCS3//TGFBR2//TNF |
| hsa05170 | Human immunodeficiency virus 1 infection - Homo sapiens (human) | http://www.genome.jp/kegg-bin/show\_pathway?hsa05170+8905+805+916+1073+1399+8065+2776+3439+51135+3725+5594+56924+5290+5291+5534+5535+5894+6500+29110+7124 | 5.800975e-03 | 20 | 404 | 212 | 7906 | 4.212013e-02 | 2.236499 | 0.049505 | AP1S2//CALM2//CD3E//CFL2//CRKL//CUL5//GNAQ//IFNA1//IRAK4//JUN//MAPK1//PAK6//PIK3CA//PIK3CB//PPP3R1//PPP3R2//RAF1//SKP1//TBK1//TNF |
| hsa05164 | Influenza A - Homo sapiens (human) | http://www.genome.jp/kegg-bin/show\_pathway?hsa05164+6347+2033+3439+3458+51135+23633+5594+57506+56001+728343+5290+5291+5894+293+9021+29110+7124 | 5.992471e-03 | 17 | 404 | 170 | 7906 | 4.258479e-02 | 2.222394 | 0.042079 | CCL2//EP300//IFNA1//IFNG//IRAK4//KPNA6//MAPK1//MAVS//NXF2//NXF2B//PIK3CA//PIK3CB//RAF1//SLC25A6//SOCS3//TBK1//TNF |
| hsa01521 | EGFR tyrosine kinase inhibitor resistance - Homo sapiens (human) | http://www.genome.jp/kegg-bin/show\_pathway?hsa01521+3479+3791+5594+4233+3084+5290+5291+5728+5894+6655 | 6.644703e-03 | 10 | 404 | 79 | 7906 | 4.623606e-02 | 2.177524 | 0.024752 | IGF1//KDR//MAPK1//MET//NRG1//PIK3CA//PIK3CB//PTEN//RAF1//SOS2 |
| hsa04722 | Neurotrophin signaling pathway - Homo sapiens (human) | http://www.genome.jp/kegg-bin/show\_pathway?hsa04722+805+1399+51135+3725+5594+4908+5290+5291+5894+5906+5908+9252+6655 | 7.524607e-03 | 13 | 404 | 119 | 7906 | 5.129018e-02 | 2.123516 | 0.032178 | CALM2//CRKL//IRAK4//JUN//MAPK1//NTF3//PIK3CA//PIK3CB//RAF1//RAP1A//RAP1B//RPS6KA5//SOS2 |
| hsa05166 | Human T-cell leukemia virus 1 infection - Homo sapiens (human) | http://www.genome.jp/kegg-bin/show\_pathway?hsa05166+109+595+894+916+2033+2113+8061+3558+3725+5594+5290+5291+5534+5535+5567+5728+5925+293+7048+7124 | 8.249633e-03 | 20 | 404 | 219 | 7906 | 5.510755e-02 | 2.083565 | 0.049505 | ADCY3//CCND1//CCND2//CD3E//EP300//ETS1//FOSL1//IL2//JUN//MAPK1//PIK3CA//PIK3CB//PPP3R1//PPP3R2//PRKACB//PTEN//RB1//SLC25A6//TGFBR2//TNF |
| hsa05213 | Endometrial cancer - Homo sapiens (human) | http://www.genome.jp/kegg-bin/show\_pathway?hsa05213+595+1647+5594+5290+5291+5728+5894+6655 | 8.808507e-03 | 8 | 404 | 58 | 7906 | 5.768709e-02 | 2.055098 | 0.019802 | CCND1//GADD45A//MAPK1//PIK3CA//PIK3CB//PTEN//RAF1//SOS2 |
| hsa04370 | VEGF signaling pathway - Homo sapiens (human) | http://www.genome.jp/kegg-bin/show\_pathway?hsa04370+3791+5594+5290+5291+5534+5535+5894+56848 | 9.749709e-03 | 8 | 404 | 59 | 7906 | 6.262313e-02 | 2.011008 | 0.019802 | KDR//MAPK1//PIK3CA//PIK3CB//PPP3R1//PPP3R2//RAF1//SPHK2 |
| hsa04960 | Aldosterone-regulated sodium reabsorption - Homo sapiens (human) | http://www.genome.jp/kegg-bin/show\_pathway?hsa04960+3479+5594+4306+5290+5291+6446 | 1.036566e-02 | 6 | 404 | 37 | 7906 | 6.532322e-02 | 1.984403 | 0.014851 | IGF1//MAPK1//NR3C2//PIK3CA//PIK3CB//SGK1 |
| hsa05162 | Measles - Homo sapiens (human) | http://www.genome.jp/kegg-bin/show\_pathway?hsa05162+595+894+916+3439+3558+51135+3725+57506+4478+5290+5291+51209+29110+7128 | 1.068526e-02 | 14 | 404 | 138 | 7906 | 6.609034e-02 | 1.971215 | 0.034653 | CCND1//CCND2//CD3E//IFNA1//IL2//IRAK4//JUN//MAVS//MSN//PIK3CA//PIK3CB//RAB9B//TBK1//TNFAIP3 |
| hsa04668 | TNF signaling pathway - Homo sapiens (human) | http://www.genome.jp/kegg-bin/show\_pathway?hsa04668+6347+1051+1906+3659+3725+5594+5290+5291+9252+9021+7124+7128 | 1.162556e-02 | 12 | 404 | 112 | 7906 | 7.059887e-02 | 1.934586 | 0.029703 | CCL2//CEBPB//EDN1//IRF1//JUN//MAPK1//PIK3CA//PIK3CB//RPS6KA5//SOCS3//TNF//TNFAIP3 |
| hsa04933 | AGE-RAGE signaling pathway in diabetic complications - Homo sapiens (human) | http://www.genome.jp/kegg-bin/show\_pathway?hsa04933+6347+595+1906+2152+3725+5594+5290+5291+7048+7124+7423 | 1.276733e-02 | 11 | 404 | 100 | 7906 | 7.546298e-02 | 1.893900 | 0.027228 | CCL2//CCND1//EDN1//F3//JUN//MAPK1//PIK3CA//PIK3CB//TGFBR2//TNF//VEGFB |
| hsa04340 | Hedgehog signaling pathway - Homo sapiens (human) | http://www.genome.jp/kegg-bin/show\_pathway?hsa04340+595+894+2121+2737+5567+57154+339745 | 1.287841e-02 | 7 | 404 | 50 | 7906 | 7.546298e-02 | 1.890138 | 0.017327 | CCND1//CCND2//EVC//GLI3//PRKACB//SMURF1//SPOPL |
| hsa04971 | Gastric acid secretion - Homo sapiens (human) | http://www.genome.jp/kegg-bin/show\_pathway?hsa04971+109+805+2776+3766+3759+54207+3776+5567+115111 | 1.371623e-02 | 9 | 404 | 75 | 7906 | 7.764782e-02 | 1.862765 | 0.022277 | ADCY3//CALM2//GNAQ//KCNJ10//KCNJ2//KCNK10//KCNK2//PRKACB//SLC26A7 |
| hsa05212 | Pancreatic cancer - Homo sapiens (human) | http://www.genome.jp/kegg-bin/show\_pathway?hsa05212+595+1647+5594+5290+5291+5894+10928+5925+7048 | 1.371623e-02 | 9 | 404 | 75 | 7906 | 7.764782e-02 | 1.862765 | 0.022277 | CCND1//GADD45A//MAPK1//PIK3CA//PIK3CB//RAF1//RALBP1//RB1//TGFBR2 |
| hsa05235 | PD-L1 expression and PD-1 checkpoint pathway in cancer - Homo sapiens (human) | http://www.genome.jp/kegg-bin/show\_pathway?hsa05235+916+3458+3725+5594+5290+5291+5534+5535+5728+5894 | 1.495359e-02 | 10 | 404 | 89 | 7906 | 8.324168e-02 | 1.825254 | 0.024752 | CD3E//IFNG//JUN//MAPK1//PIK3CA//PIK3CB//PPP3R1//PPP3R2//PTEN//RAF1 |
| hsa04625 | C-type lectin receptor signaling pathway - Homo sapiens (human) | http://www.genome.jp/kegg-bin/show\_pathway?hsa04625+805+3558+3659+3725+5594+5290+5291+5534+5535+5894+7124 | 1.678100e-02 | 11 | 404 | 104 | 7906 | 9.188285e-02 | 1.775182 | 0.027228 | CALM2//IL2//IRF1//JUN//MAPK1//PIK3CA//PIK3CB//PPP3R1//PPP3R2//RAF1//TNF |
| hsa04071 | Sphingolipid signaling pathway - Homo sapiens (human) | http://www.genome.jp/kegg-bin/show\_pathway?hsa04071+2776+5594+5290+5291+5515+5516+5529+5728+5894+1901+56848+7124 | 1.816578e-02 | 12 | 404 | 119 | 7906 | 9.615518e-02 | 1.740746 | 0.029703 | GNAQ//MAPK1//PIK3CA//PIK3CB//PPP2CA//PPP2CB//PPP2R5E//PTEN//RAF1//S1PR1//SPHK2//TNF |
| hsa04658 | Th1 and Th2 cell differentiation - Homo sapiens (human) | http://www.genome.jp/kegg-bin/show\_pathway?hsa04658+916+28514+3458+3558+3725+4094+5594+5534+5535+864 | 1.853629e-02 | 10 | 404 | 92 | 7906 | 9.615518e-02 | 1.731977 | 0.024752 | CD3E//DLL1//IFNG//IL2//JUN//MAF//MAPK1//PPP3R1//PPP3R2//RUNX3 |
| hsa05223 | Non-small cell lung cancer - Homo sapiens (human) | http://www.genome.jp/kegg-bin/show\_pathway?hsa05223+595+1647+5594+5290+5291+5894+5925+6655 | 1.855258e-02 | 8 | 404 | 66 | 7906 | 9.615518e-02 | 1.731596 | 0.019802 | CCND1//GADD45A//MAPK1//PIK3CA//PIK3CB//RAF1//RB1//SOS2 |
| hsa03018 | RNA degradation - Homo sapiens (human) | http://www.genome.jp/kegg-bin/show\_pathway?hsa03018+694+57472+246175+29883+167227+1656+255967+219988+10140 | 1.881509e-02 | 9 | 404 | 79 | 7906 | 9.615518e-02 | 1.725494 | 0.022277 | BTG1//CNOT6//CNOT6L//CNOT7//DCP2//DDX6//PAN3//PATL1//TOB1 |
| hsa04152 | AMPK signaling pathway - Homo sapiens (human) | http://www.genome.jp/kegg-bin/show\_pathway?hsa04152+79602+595+3479+5290+5291+5468+5515+5516+5529+5562+23411+7248 | 1.928861e-02 | 12 | 404 | 120 | 7906 | 9.615518e-02 | 1.714699 | 0.029703 | ADIPOR2//CCND1//IGF1//PIK3CA//PIK3CB//PPARG//PPP2CA//PPP2CB//PPP2R5E//PRKAA1//SIRT1//TSC1 |
| hsa05135 | Yersinia infection - Homo sapiens (human) | http://www.genome.jp/kegg-bin/show\_pathway?hsa05135+6347+1399+9815+3558+51135+3725+5594+5290+5291+8935+29110+7124 | 1.928861e-02 | 12 | 404 | 120 | 7906 | 9.615518e-02 | 1.714699 | 0.029703 | CCL2//CRKL//GIT2//IL2//IRAK4//JUN//MAPK1//PIK3CA//PIK3CB//SKAP2//TBK1//TNF |
| hsa04657 | IL-17 signaling pathway - Homo sapiens (human) | http://www.genome.jp/kegg-bin/show\_pathway?hsa04657+6347+1051+8061+3458+3725+5594+5597+29110+7124+7128 | 1.986020e-02 | 10 | 404 | 93 | 7906 | 9.754863e-02 | 1.702016 | 0.024752 | CCL2//CEBPB//FOSL1//IFNG//JUN//MAPK1//MAPK6//TBK1//TNF//TNFAIP3 |
| hsa05163 | Human cytomegalovirus infection - Homo sapiens (human) | http://www.genome.jp/kegg-bin/show\_pathway?hsa05163+109+805+6347+595+1399+2776+3439+5594+5290+5291+5534+5535+5567+5894+5925+6655+29110+7124+7248 | 2.123145e-02 | 19 | 404 | 225 | 7906 | 1.027725e-01 | 1.673020 | 0.047030 | ADCY3//CALM2//CCL2//CCND1//CRKL//GNAQ//IFNA1//MAPK1//PIK3CA//PIK3CB//PPP3R1//PPP3R2//PRKACB//RAF1//RB1//SOS2//TBK1//TNF//TSC1 |
| hsa05031 | Amphetamine addiction - Homo sapiens (human) | http://www.genome.jp/kegg-bin/show\_pathway?hsa05031+23237+805+3725+84152+5534+5535+5567+23411 | 2.187102e-02 | 8 | 404 | 68 | 7906 | 1.029403e-01 | 1.660131 | 0.019802 | ARC//CALM2//JUN//PPP1R1B//PPP3R1//PPP3R2//PRKACB//SIRT1 |
| hsa04136 | Autophagy - other - Homo sapiens (human) | http://www.genome.jp/kegg-bin/show\_pathway?hsa04136+55054+23710+5515+5516+9706 | 2.188251e-02 | 5 | 404 | 32 | 7906 | 1.029403e-01 | 1.659903 | 0.012376 | ATG16L1//GABARAPL1//PPP2CA//PPP2CB//ULK2 |
| hsa04910 | Insulin signaling pathway - Homo sapiens (human) | http://www.genome.jp/kegg-bin/show\_pathway?hsa04910+805+1399+5594+5290+5291+5562+5567+5577+5894+8651+9021+6655+7248 | 2.260928e-02 | 13 | 404 | 137 | 7906 | 1.048819e-01 | 1.645713 | 0.032178 | CALM2//CRKL//MAPK1//PIK3CA//PIK3CB//PRKAA1//PRKACB//PRKAR2B//RAF1//SOCS1//SOCS3//SOS2//TSC1 |
| hsa04962 | Vasopressin-regulated water reabsorption - Homo sapiens (human) | http://www.genome.jp/kegg-bin/show\_pathway?hsa04962+109+84516+10671+1783+140735+5567 | 2.335262e-02 | 6 | 404 | 44 | 7906 | 1.058587e-01 | 1.631664 | 0.014851 | ADCY3//DCTN5//DCTN6//DYNC1LI2//DYNLL2//PRKACB |
| hsa04662 | B cell receptor signaling pathway - Homo sapiens (human) | http://www.genome.jp/kegg-bin/show\_pathway?hsa04662+1380+3725+5594+5290+5291+5534+5535+5894+6655 | 2.345372e-02 | 9 | 404 | 82 | 7906 | 1.058587e-01 | 1.629788 | 0.022277 | CR2//JUN//MAPK1//PIK3CA//PIK3CB//PPP3R1//PPP3R2//RAF1//SOS2 |
| hsa04921 | Oxytocin signaling pathway - Homo sapiens (human) | http://www.genome.jp/kegg-bin/show\_pathway?hsa04921+109+805+595+2776+3725+3759+3760+5594+4659+5534+5535+5562+5567+5894 | 2.439382e-02 | 14 | 404 | 153 | 7906 | 1.086338e-01 | 1.612720 | 0.034653 | ADCY3//CALM2//CCND1//GNAQ//JUN//KCNJ2//KCNJ3//MAPK1//PPP1R12A//PPP3R1//PPP3R2//PRKAA1//PRKACB//RAF1 |
| hsa05169 | Epstein-Barr virus infection - Homo sapiens (human) | http://www.genome.jp/kegg-bin/show\_pathway?hsa05169+595+894+916+1380+1647+3439+51135+3725+57506+5290+5291+5718+5925+864+29110+7124+7128 | 2.797130e-02 | 17 | 404 | 201 | 7906 | 1.229265e-01 | 1.553287 | 0.042079 | CCND1//CCND2//CD3E//CR2//GADD45A//IFNA1//IRAK4//JUN//MAVS//PIK3CA//PIK3CB//PSMD12//RB1//RUNX3//TBK1//TNF//TNFAIP3 |
| hsa04930 | Type II diabetes mellitus - Homo sapiens (human) | http://www.genome.jp/kegg-bin/show\_pathway?hsa04930+5594+5290+5291+8651+9021+7124 | 2.846762e-02 | 6 | 404 | 46 | 7906 | 1.234829e-01 | 1.545649 | 0.014851 | MAPK1//PIK3CA//PIK3CB//SOCS1//SOCS3//TNF |
| hsa04914 | Progesterone-mediated oocyte maturation - Homo sapiens (human) | http://www.genome.jp/kegg-bin/show\_pathway?hsa04914+109+64506+132864+22849+3479+5594+5290+5291+5567+5894 | 2.929231e-02 | 10 | 404 | 99 | 7906 | 1.254312e-01 | 1.533246 | 0.024752 | ADCY3//CPEB1//CPEB2//CPEB3//IGF1//MAPK1//PIK3CA//PIK3CB//PRKACB//RAF1 |
| hsa05165 | Human papillomavirus infection - Homo sapiens (human) | http://www.genome.jp/kegg-bin/show\_pathway?hsa05165+595+894+2033+7976+8323+3439+3659+3696+5594+5290+5291+5515+5516+5529+5567+5728+5894+5925+6655+29110+7124+7248+80326+7473+7476 | 3.072372e-02 | 25 | 404 | 330 | 7906 | 1.298952e-01 | 1.512526 | 0.061881 | CCND1//CCND2//EP300//FZD3//FZD6//IFNA1//IRF1//ITGB8//MAPK1//PIK3CA//PIK3CB//PPP2CA//PPP2CB//PPP2R5E//PRKACB//PTEN//RAF1//RB1//SOS2//TBK1//TNF//TSC1//WNT10A//WNT3//WNT7A |
| hsa04926 | Relaxin signaling pathway - Homo sapiens (human) | http://www.genome.jp/kegg-bin/show\_pathway?hsa04926+109+1906+1910+3725+5594+5290+5291+5567+5894+6655+7048+7423 | 3.184425e-02 | 12 | 404 | 129 | 7906 | 1.329497e-01 | 1.496969 | 0.029703 | ADCY3//EDN1//EDNRB//JUN//MAPK1//PIK3CA//PIK3CB//PRKACB//RAF1//SOS2//TGFBR2//VEGFB |
| hsa04730 | Long-term depression - Homo sapiens (human) | http://www.genome.jp/kegg-bin/show\_pathway?hsa04730+1394+2776+3479+5594+5515+5516+5894 | 3.230560e-02 | 7 | 404 | 60 | 7906 | 1.332107e-01 | 1.490722 | 0.017327 | CRHR1//GNAQ//IGF1//MAPK1//PPP2CA//PPP2CB//RAF1 |
| hsa04540 | Gap junction - Homo sapiens (human) | http://www.genome.jp/kegg-bin/show\_pathway?hsa04540+109+153+2697+2776+1902+5594+5567+5894+6655 | 3.507730e-02 | 9 | 404 | 88 | 7906 | 1.428758e-01 | 1.454974 | 0.022277 | ADCY3//ADRB1//GJA1//GNAQ//LPAR1//MAPK1//PRKACB//RAF1//SOS2 |
| hsa04630 | JAK-STAT signaling pathway - Homo sapiens (human) | http://www.genome.jp/kegg-bin/show\_pathway?hsa04630+595+894+1271+2033+3439+3458+3558+5290+5291+5894+8651+9021+9655+6655 | 3.729287e-02 | 14 | 404 | 162 | 7906 | 1.479079e-01 | 1.428374 | 0.034653 | CCND1//CCND2//CNTFR//EP300//IFNA1//IFNG//IL2//PIK3CA//PIK3CB//RAF1//SOCS1//SOCS3//SOCS5//SOS2 |
| hsa05161 | Hepatitis B - Homo sapiens (human) | http://www.genome.jp/kegg-bin/show\_pathway?hsa05161+2033+3439+51135+3725+5594+57506+5290+5291+5894+5925+6655+29110+7048+7124 | 3.729287e-02 | 14 | 404 | 162 | 7906 | 1.479079e-01 | 1.428374 | 0.034653 | EP300//IFNA1//IRAK4//JUN//MAPK1//MAVS//PIK3CA//PIK3CB//RAF1//RB1//SOS2//TBK1//TGFBR2//TNF |
| hsa04213 | Longevity regulating pathway - multiple species - Homo sapiens (human) | http://www.genome.jp/kegg-bin/show\_pathway?hsa04213+109+3479+5290+5291+5562+5567+23411 | 3.774835e-02 | 7 | 404 | 62 | 7906 | 1.479079e-01 | 1.423102 | 0.017327 | ADCY3//IGF1//PIK3CA//PIK3CB//PRKAA1//PRKACB//SIRT1 |
| hsa00603 | Glycosphingolipid biosynthesis - globo and isoglobo series - Homo sapiens (human) | http://www.genome.jp/kegg-bin/show\_pathway?hsa00603+2524+10690+4668 | 3.810545e-02 | 3 | 404 | 15 | 7906 | 1.479079e-01 | 1.419013 | 0.007426 | FUT2//FUT9//NAGA |
| hsa05216 | Thyroid cancer - Homo sapiens (human) | http://www.genome.jp/kegg-bin/show\_pathway?hsa05216+8030+595+1647+5594+5468 | 3.852692e-02 | 5 | 404 | 37 | 7906 | 1.479079e-01 | 1.414236 | 0.012376 | CCDC6//CCND1//GADD45A//MAPK1//PPARG |
| hsa04072 | Phospholipase D signaling pathway - Homo sapiens (human) | http://www.genome.jp/kegg-bin/show\_pathway?hsa04072+109+8526+160851+2913+1902+5594+5290+5291+5894+11069+6655+56848+7248 | 3.915640e-02 | 13 | 404 | 148 | 7906 | 1.486163e-01 | 1.407197 | 0.032178 | ADCY3//DGKE//DGKH//GRM3//LPAR1//MAPK1//PIK3CA//PIK3CB//RAF1//RAPGEF4//SOS2//SPHK2//TSC1 |
| hsa05217 | Basal cell carcinoma - Homo sapiens (human) | http://www.genome.jp/kegg-bin/show\_pathway?hsa05217+7976+8323+1647+2737+80326+7473+7476 | 4.068261e-02 | 7 | 404 | 63 | 7906 | 1.521530e-01 | 1.390591 | 0.017327 | FZD3//FZD6//GADD45A//GLI3//WNT10A//WNT3//WNT7A |
| hsa04261 | Adrenergic signaling in cardiomyocytes - Homo sapiens (human) | http://www.genome.jp/kegg-bin/show\_pathway?hsa04261+109+153+805+2776+3753+5594+5515+5516+5529+5567+11069+9252+6324 | 4.099931e-02 | 13 | 404 | 149 | 7906 | 1.521530e-01 | 1.387223 | 0.032178 | ADCY3//ADRB1//CALM2//GNAQ//KCNE1//MAPK1//PPP2CA//PPP2CB//PPP2R5E//PRKACB//RAPGEF4//RPS6KA5//SCN1B |
| hsa04810 | Regulation of actin cytoskeleton - Homo sapiens (human) | http://www.genome.jp/kegg-bin/show\_pathway?hsa04810+50649+1073+1129+1399+55740+10788+3696+1902+5594+4478+56924+5217+5290+5291+4659+5894+6655 | 4.488839e-02 | 17 | 404 | 213 | 7906 | 1.647552e-01 | 1.347866 | 0.042079 | ARHGEF4//CFL2//CHRM2//CRKL//ENAH//IQGAP2//ITGB8//LPAR1//MAPK1//MSN//PAK6//PFN2//PIK3CA//PIK3CB//PPP1R12A//RAF1//SOS2 |
| hsa04666 | Fc gamma R-mediated phagocytosis - Homo sapiens (human) | http://www.genome.jp/kegg-bin/show\_pathway?hsa04666+1073+1399+5594+4082+5290+5291+5894+56848+10810 | 4.739057e-02 | 9 | 404 | 93 | 7906 | 1.715555e-01 | 1.324308 | 0.022277 | CFL2//CRKL//MAPK1//MARCKS//PIK3CA//PIK3CB//RAF1//SPHK2//WASF3 |
| hsa00983 | Drug metabolism - other enzymes - Homo sapiens (human) | http://www.genome.jp/kegg-bin/show\_pathway?hsa00983+51727+2939+2947+3251+3614+4257+54490+7364 | 4.776845e-02 | 8 | 404 | 79 | 7906 | 1.715555e-01 | 1.320859 | 0.019802 | CMPK1//GSTA2//GSTM3//HPRT1//IMPDH1//MGST1//UGT2B28//UGT2B7 |
| hsa04915 | Estrogen signaling pathway - Homo sapiens (human) | http://www.genome.jp/kegg-bin/show\_pathway?hsa04915+109+805+2099+2776+3725+3760+5594+5290+5291+5567+5894+6655 | 4.940922e-02 | 12 | 404 | 138 | 7906 | 1.755604e-01 | 1.306192 | 0.029703 | ADCY3//CALM2//ESR1//GNAQ//JUN//KCNJ3//MAPK1//PIK3CA//PIK3CB//PRKACB//RAF1//SOS2 |
